# Heat Shock Transcription Factor 1-Deficiency Attenuates Overloading-Associated Hypertrophy of Mouse Soleus Muscle

**DOI:** 10.1371/journal.pone.0077788

**Published:** 2013-10-22

**Authors:** Tomoyuki Koya, Sono Nishizawa, Yoshitaka Ohno, Ayumi Goto, Akihiro Ikuta, Miho Suzuki, Tomotaka Ohira, Tatsuro Egawa, Akira Nakai, Takao Sugiura, Yoshinobu Ohira, Toshitada Yoshioka, Moroe Beppu, Katsumasa Goto

**Affiliations:** 1 Department of Orthopaedic Surgery, St. Marianna University School of Medicine, Kawasaki, Kanagawa, Japan; 2 Laboratory of Physiology, School of Health Science, Toyohashi SOZO University, Toyohashi, Aichi, Japan; 3 Department of Physiology, Graduate School of Health Science, Toyohashi SOZO University, Toyohashi, Aichi, Japan; 4 Department of Molecular Biology, Graduate School of Medicine, Yamaguchi University, Ube, Yamaguchi, Japan; 5 Department of Exercise and Health Sciences, Yamaguchi University, Yamaguchi City, Yamaguchi, Japan; 6 Graduate School of Medicine, Osaka University, Toyonaka, Osaka, Japan; 7 Hirosaki Gakuin University, Hirosaki, Aomori, Japan; University of Minnesota Medical School, United States of America

## Abstract

Hypertrophic stimuli, such as mechanical stress and overloading, induce stress response, which is mediated by heat shock transcription factor 1 (HSF1), and up-regulate heat shock proteins (HSPs) in mammalian skeletal muscles. Therefore, HSF1-associated stress response may play a key role in loading-associated skeletal muscle hypertrophy. The purpose of this study was to investigate the effects of HSF1-deficiency on skeletal muscle hypertrophy caused by overloading. Functional overloading on the left soleus was performed by cutting the distal tendons of gastrocnemius and plantaris muscles for 4 weeks. The right muscle served as the control. Soleus muscles from both hindlimbs were dissected 2 and 4 weeks after the operation. Hypertrophy of soleus muscle in HSF1-null mice was partially inhibited, compared with that in wild-type (C57BL/6J) mice. Absence of HSF1 partially attenuated the increase of muscle wet weight and fiber cross-sectional area of overloaded soleus muscle. Population of Pax7-positive muscle satellite cells in HSF1-null mice was significantly less than that in wild-type mice following 2 weeks of overloading (p<0.05). Significant up-regulations of interleukin (IL)-1β and tumor necrosis factor mRNAs were observed in HSF1-null, but not in wild-type, mice following 2 weeks of overloading. Overloading-related increases of IL-6 and AFT3 mRNA expressions seen after 2 weeks of overloading tended to decrease after 4 weeks in both types of mice. In HSF1-null mice, however, the significant overloading-related increase in the expression of IL-6, not ATF3, mRNA was noted even at 4th week. Inhibition of muscle hypertrophy might be attributed to the greater and prolonged enhancement of IL-6 expression. HSF1 and/or HSF1-mediated stress response may, in part, play a key role in loading-induced skeletal muscle hypertrophy.

## Introduction

Mechanical loading is one of the important factors in the regulation of skeletal muscle size. Skeletal muscle is highly plastic and adapts to physical demand. Increase in the mechanical load on skeletal muscle causes hypertrophy, whereas unloading induces atrophy. It has been generally accepted that increased loading activates muscle satellite cells, which are skeletal muscle-specific stem cells, and stimulates muscle protein synthesis. Hypertrophied muscle fibers, caused by mechanical loading, have a larger diameter, greater protein content, and increased number of myonuclei compared with sedentary control fibers. Although loading-dependent muscle hypertrophy is attributed to both the activation of muscle satellite cells and the stimulation of protein synthesis [1], [2], the mechanisms responsible for these systems are not fully elucidated.

Stress proteins, so-called heat shock proteins (HSPs), are up-regulated by hypertrophic stimuli, such as mechanical stretch, loading, and heat stress [3], [4]. HSPs, which act as the molecular chaperones, play a part of the tightly regulated systems for maintenance of cellular homeostasis for survival in response to various pathological conditions [5], [6]. HSPs, especially inducible 70 kDa HSP (HSP70, so-called HSP72), as well as HSP25, are induced and protect against cellular stresses via so-called stress response [3], [4], [7]–[9]. Since HSP25 and HSP72 function as important molecular chaperones [3], [4], [8], there are many reports showing the prevention of disuse- and/or immobilization-associated skeletal muscle atrophy by overexpression of HSP25 or HSP72 [10], [11]. Previous studies also demonstrated up-regulations of HSP25, HSP72 and/or HSP90 in hypertrophied skeletal muscles of rats [12]–[15] and mice [13]. However, physiological role(s) of up-regulation of HSPs for induction of hypertrophy of skeletal muscle cells remains unclear.

Since HSP25 and HSP72 are up-regulated during regrowth of mouse soleus muscle from unloading-related atrophy [16], it is suggested that these proteins may play role(s) in overloading-associated skeletal muscle hypertrophy. HSP47, which is known as a collagen specific HSP [17], in anti-gravitational rat soleus muscle is up-regulated by hypergravity [18]. However, it is still unknown whether HSF1-deficiency influences the expression levels of HSP25, HSP47, HSP72, and HSP90 during skeletal muscle hypertrophy.

Heat shock transcription factors (HSFs), which mediate stress response, up-regulate the expression of HSPs via binding to heat shock element, located at the up-stream region of HSP genes [19], [20]. Among three HSFs (HSF1, HSF2, and HSF4) in mammals, HSF1 plays a crucial role in inducing HSPs, conferring cytoprotection against various stresses [21], [22]. In the previous study, we observed that HSF1-deficiency retarded the regrowth of atrophied solues muscles, which are induced by hindlimb unloading [16]. Recently, we have also reported that HSF1-deficiency did not affect the population level of Pax7-positive satellite cells in the untreated control muscle [23]. However, effects of HSF1-deficiency on the loading-associated skeletal muscle hypertrophy are still unknown.

HSF1 suppresses inflammatory genes, including interleukin-6 (IL-6), through activating transcription factor 3 (ATF3) [24]. On the contrary, it has been suggested that HSF1 and HSF2 activate IL-6 synthesis [25]. IL-6, which is well-known as a pleiotropic cytokine, associated with the regulation and coordination of immune response [26], and other pro-inflammatory cytokines, such as IL-1β and tumor necrosis factor (TNF), are potentially mitogenic for myoblasts, as well as inhibitors of myogenic differentiation [27]–[29]. Intracellular signaling of TNFα, one of TNF superfamily, impairs insulin/insulin-like growth factor signaling by insulin receptor substrate-1 and Akt (so-called protein kinase B: PKB) [30], [31], which plays a key role in skeletal muscle hypertrophy [32]. These observations suggest that HSF1 may modulate skeletal muscle hypertrophy via the behavior of IL-6-mediated muscle satellite cells. However, the effects of HSF1-deficiency on the responses of pro-inflammatory cytokines, as well as Akt, during overloading-associated skeletal muscle hypertrophy are unclear. Therefore, the current study was performed to investigate the physiological role of HSF1 gene on overloading-associated skeletal muscle hypertrophy by using the HSF1-null mice.

## Materials and Methods

### Animals

All animal protocols were carried out in accordance with the Guide for the Care and Use of Laboratory Animals as adopted and promulgated by the National Institutes of Health (Bethesda, MD) and were approved by the Animal Use Committee at Toyohashi SOZO University (2007001). All treatments for animals were performed under anesthesia with *i.p.* injection of sodium pentobarbital, and all efforts were made to prevent discomfort and suffering. Male HSF1-null and wild-type (ICR) mice were prepared as described previously [16]. Mice with 10–15 wk of age were used in this experiment (n  =  12 in each type of mice). Two or three mice were housed in a cage (20×31 cm and 13.5 cm height). All mice were housed in a vivarium room with 12:12-h light:dark cycle and 23°C and ∼50% temperature and humidity, respectively. Solid food and water were provided *ad libitum*.

### Functional overloading

Functional overloading on the left soleus was unilaterally performed by removal of the distal tendons of both plantaris and gastrocnemius muscles under anesthesia with intrapenitoneal injection of pentobarbital sodium as described earlier [2]. The contralateral right muscle received sham operation as the control. The muscle was kept intact, although it was exposed surgically. The skin was sutured and mice were housed in the cages. It has been reported that unilateral functional overloading does not affect the myosin heavy chain (MHC) gene expression in the contralateral muscle, and the locomortion and mobility were unaltered by the unilateral functional overloading within 1 day post-surgery [12].

### Sampling

Soleus muscles were dissected from the both hindlimbs 2 and 4 weeks after the surgery. All muscles were rapidly weighed (wet weight) and divided into three portions cross-sectionally. Then, muscle sections were frozen in liquid nitrogen and stored at −80°C until analyzed.

### Muscle protein content

The middle portions of muscles were homogenized in ∼0.4 ml (0.1 ml/mg muscle wet wt) of tissue lysis reagent (CelLytic-MT, Sigma-Aldrich, St. Louis, MO) and completely solubilized by alkaline treatment with 2 N NaOH at 37°C for 1 h [16]. Protein concentration of the tissue lysate was determined by using protein assay kit (Bio-Rad, Hercules, CA) and bovine serum albumin (Sigma) as the standard. Total protein content in whole muscle was then calculated.

### Real-time reverse transcription-polymerase chain reaction (Real-time RT-PCR)

In the present study, the expressions of HSP mRNAs, including HSP25, HSP47, the constitutive cytosolic HSC70, so-called HSP73, stress inducible HSP72, and HSP90α, and the expressions of HSF mRNAs, including HSF1, HSF2, and HSF4, were assessed by real-time RT-PCR. Pro-inflammatory cytokine mRNAs, such as IL-6, ATF3, IL-1β, and TNF, were also evaluated. Total RNA was extracted from the proximal portion of muscle using the miRNeasy Mini kit (Qiagen, Hiden, Germany) according to the manufacturer’s protocol. Samples (∼40 ng of RNA) were reverse-transcribed using the first strand cDNA synthesis kit according to the manufacturer’s instructions {PrimeScript RT Master Mix (Perfect Real Time) for mRNA, Takara Bio, Otsu, Japan}. Synthesized cDNA was applied to real-time reverse transcription-PCR (Thermal Cycler Dice Real Time System II MRQ, Takara Bio) using Takara SYBR Premix Ex Taq II for mRNA, and analyzed using Takara Thermal Cycler Dice Real Time System Software (version 4.00) according to the manufacturer’s instructions. The real-time cycle conditions were 95°C for 30 s followed by 40 cycles at 95°C for 5 s and at 60°C for 30 s for mRNA. Specificity was confirmed by electrophoretic analysis of the reaction products and by inclusion of template- or reverse transcriptase-free controls. To normalize the amount of total RNA present in each reaction, glyceraldehyde-3-phosphate dehydrogenase (GAPDH) was used as an internal standard.

The primers were designed by using the Takara Bio Perfect Real Time Support System (Takara Bio). Primers used for detection of mouse cDNA were as follows: HSP25, 5′-TCCCTGGACGTCAACCACTTC-3′ (forward) and 5′-AGAGATGTAGCCATGTTCGTCCTG-3′ (reverse); HSP47, 5′-TGAGGTCACCAAGGATGTGTGGAG-3′ (forward) and 5′-AGGAGCGGGTCACCATGAAG -3′ (reverse); HSC70, 5′-AGCTGCCTGGCATTTGTGTG-3′ (forward) and 5′-GTGCGGTTACCCTGGTCATTG-3′ (reverse); HSP72, 5′-CAAGAACGCGCTCGAATCCTA-3′ (forward) and 5′-TCCTGGCACTTGTCCAGCAC-3′ (reverse); HSP90α, 5′-CCATGCTAACAGGATCTACAGGA-3′ (forward) and 5′-TCTTCAGTTACAGCAGCACTGG-3′ (reverse); HSF1, 5′-ACAGTGTCACCCGGCTGTTG-3′ (forward) and 5′-GACTGCACCAGTGAGATGAGGAA-3′ (reverse); HSF2, 5′-GCAGTGTTGTTCAACATGTGTCAG-3′ (forward) and 5′-AGTTCCCATCCAGGAATGCAAG-3′ (reverse); HSF4, 5′-TGATGGATCTGGACATGGAGTTG-3′ (forward) and 5′-CTAGCATGAGTGGAGTTCCCAGTG-3′ (reverse); IL-6, 5′- CCACTTCACAAGTCGGAGGCTTA-3′ (forward) and 5′- GCAAGTGCATCATCGTTGTTCATAC-3′ (reverse); ATF3, 5′- GCTGCTGCCAAGTGTCGAA-3′ (forward) and 5′- CGGTGCAGGTTGAGCATGTATATC-3′ (reverse); IL-1β, 5′- TCCAGGATGAGGACATGAGCAC-3′ (forward) and 5′- GAACGTCACACACCAGCAGGTTA-3′ (reverse); TNF, 5′- TATGGCCCAGACCCTCACA-3′ (forward) and 5′- GGAGTAGACAAGGTACAACCCATC-3′ (reverse); GAPDH, 5′- TGTGTCCGTCGTGGATCTGA-3′ (forward) and 5′- TTGCTGTTGAAGTCGCAGGAG-3′ (reverse).

### Histochemical and immunohistochemical analyses

Serial transverse cryosections (7-µm thick) of frozen distal portion of soleus muscles were cut at −20°C and mounted on the slide glasses. The sections were air-dried and stained to analyze the degree of muscle damage and repair and the cross-sectional area (CSA) of muscle fibers by staining using hematoxylin and eosin (H&E), and the profiles of Pax7-positive nuclei by the standard immunohistochemical technique, respectively [33].

Monoclonal anti-Pax7 antibody (Developmental Studies Hybridoma Bank, Iowa, IA, USA) was used for the detection of muscle satellite cells [34]. Cross sections were fixed with paraformaldehyde (4%), and then were post-fixed in ice-cold methanol. After blocking by using a reagent (1% Roche Blocking Regent; Roche Diagonost, Penzberg, Germany), samples were incubated with the primary antibodies for Pax7 and rabbit polyclonal anti-laminin. Sections were also incubated with the second primary antibodies for Cy3-conjugated antimouse IgG1 (diluted 1:500; Jackson Immuno Research, West Grove, PA, USA) and for fluorescein isothiocyanate-conjugated anti-rabbit IgG (diluted 1:500; Sigma). Nuclei were then stained for 15 min in a solution of 4’6-diamidino-2-phenylindole dihydrochloride (DAPI, 0.5 mg/mL; Sigma). The images of muscle sections were incorporated into a personal computer (DP Manager version 2.2.1.195, Olympus Japan, Tokyo) by using a microscope (IX 81 Olympus Japan). The CSAs of ∼100 fibers from each muscle were analyzed on laminin-stained images by using ImageJ.

### Immunoblotting analyses

Expressions of HSP25, HSP47, HSC70, HSP72, HSP90α, and total and phosphorylated Akt proteins were assessed by immunoblotting assay. Proximal portions of the right muscles were homogenized in an isolation buffer of tissue lysis reagent (CelLytic-MT, Sigma-Aldrich) with 1 mM Na3VO4, 1 mM phenylmethylsulfonyl fluoride (PMSF) and 1g/ml leupeptin with glass homogenizer. The homogenates were then sonicated and centrifuged at 12,000 rpm (4°C for 10 min), and the supernatant was collected. A part of the supernatant was solubilized in sodium-dodecylsulfate (SDS) sample buffer {30% (vol/vol) glycerol, 5% (vol/vol) 2-mercaptoethanol, 2.3% (wt/vol) SDS, 62.5 mM Tris·HCl, 0.05% (wt/vol) bromophenol blue, and pH 6.8} at a concentration of 0.5 mg of protein per milliliter and boiled for 3 min. The SDS-polyacrylamide gel electrophoresis (PAGE) was carried out on 10 or 12.5% polyacrylamide {bisacrylamide/acrylamide, 1:20 (wt/wt)} slab gel (60×85×1 mm) containing 0.5% SDS at a constant current of 20 mA for 120 min, as was reported previously [35]. Equal amounts of protein (20 µg) were loaded on each gel. Molecular weight markers (ECL DualVue Western Blotting Markers, GE Healthcare, Buckinghamshire, UK) were applied to both sides of 14 lanes as the internal controls for transfer process or electrophoresis.

Following SDS-PAGE, proteins were transferred to PVDF membranes (0.2 µm pore size, Bio-Rad) at a constant voltage of 100 V for 60 min at 4°C. The membranes were blocked for 1 h using a blocking buffer: 5% skim milk with 0.1% Tween 20 in Tris-buffered saline (TTBS) with pH 7.5. The membranes were incubated for 1 h with a polyclonal antibody for HSP25 (SPA-801, StressGen, Victoria, BC), HSC70 (SPA-816, StressGen), HSP72 (SPA-812, StressGen), p-Akt (9271, Cell Signaling Technology, Beverly, Mass., USA), total Akt (9272, Cell Signaling Technology), and then reacted with a secondary antibody (goat anti-rabbit immunoglobulinG conjugate to horseradish peroxidase, Cell Signaling Technology) for 1 or 2 h. To detect HSP47 and HSP90α, antiserum for HSP47 and HSP90α were generated by immunizing rabbit, as described previously [36], [37]. After the final wash, protein bands were visualized using chemiluminescence (ECL Advance Western blotting kit, GE Healthcare), and signal density was measured using Light-Capture (AE-6971) with CS Analyzer version 2.08b (ATTO, Tokyo, Japan). The activation level of Akt was evaluated by using the phosphorylated level of Akt relative to the total expression form (p-Akt/t-Akt), since phosphorylated form of Akt is enzymatically active [38], [39]. Each sample was analyzed in duplicate, at least, to ensure that results were not influenced by loading errors. The GAPDH (G9545, Sigma-Aldrich) was evaluated to ensure the equal loading. Standard curves were constructed during the preliminary experiments to ensure linearity.

### Statistical analyses

All values were expressed as means ± SEMs. Statistical significances for body and muscle weights, protein content, expression levels of mRNA and protein were tested by using 2-way (mice and time for body weight; mice and treatments for other measurements except HSF1 mRNA) analysis of variance (ANOVA). When any significant main effects (factors) and interactions among factors were observed, Turkey-Kramer post hoc test was performed in each effect or among groups. For HSF1 mRNA, data was analyzed by using one-way ANOVA followed by Turkey-Kramer post hoc test. The significance level was accepted at p<0.05.

## Results

### Body weight and soleus muscle wet weight

**Figure 1:**
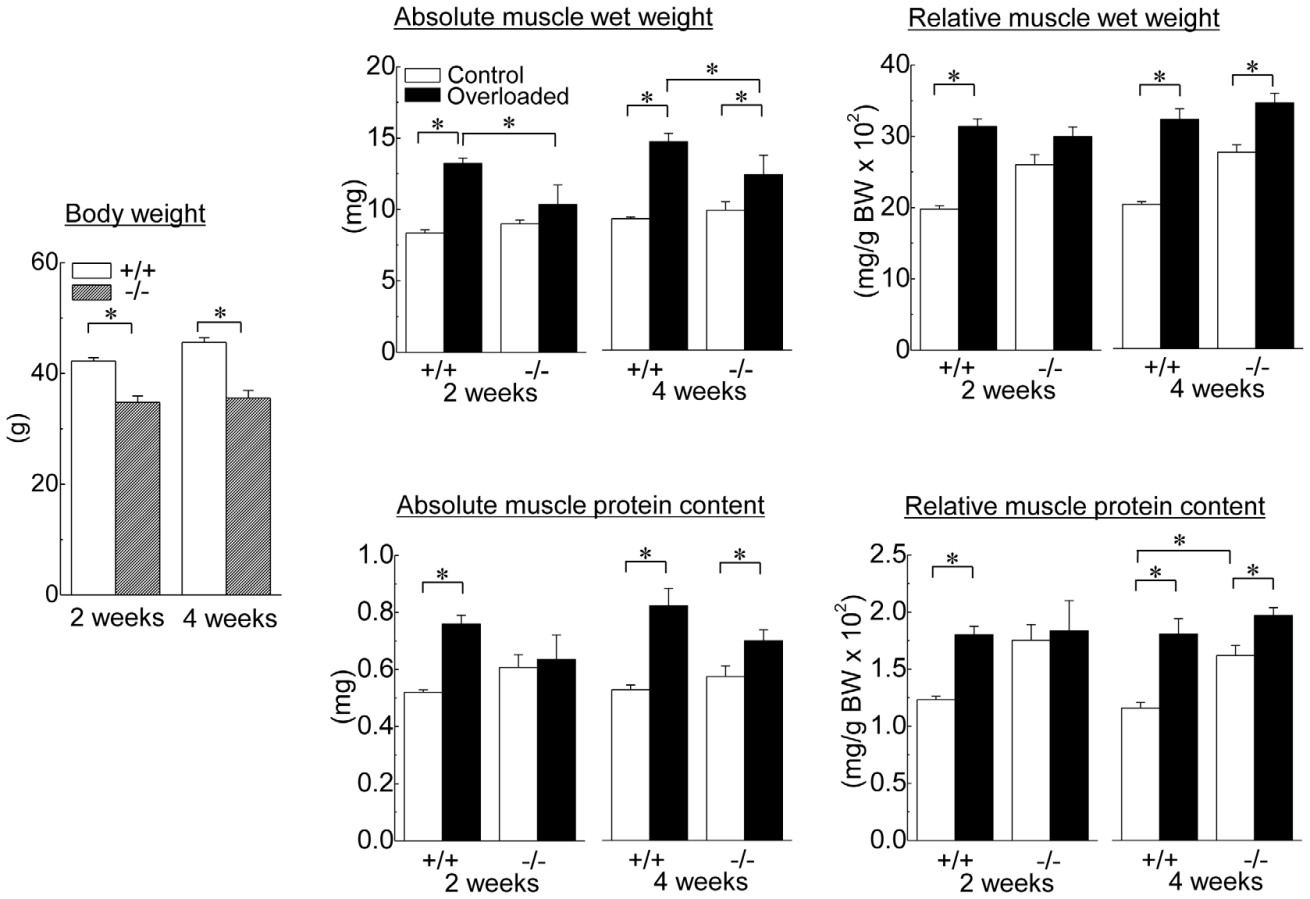
Changes in the body weight, and absolute and relative muscle weight of heat shock transcription factor 1 (HSF1)-null and wild-type mice following 2 and 4 weeks of functional overloading. Relative muscle weight: the muscle weight relative to body weight (BW); +/+, wild-type mice; −/−, HSF1-null mice; Control, control muscle; Overloaded, overloaded muscle; 2 weeks, 2 weeks of overloading; 4 weeks, 4 weeks of overloading. Values are means ± SEM. n = 6/group at each time point. *: significant difference at p<0.05.

**Figure 2:**
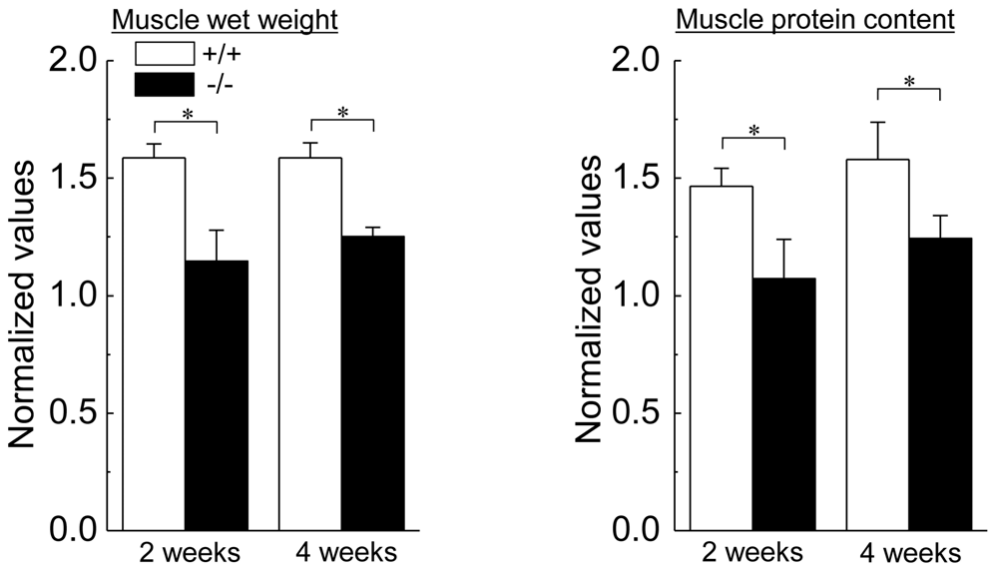
The rates of increase in the relative muscle weight and protein content to body weight. Each bar represents the relative value of overloaded muscle vs. the contralateral control muscle, which is 1.0. See figure 1 for other abbreviations. Values are means ± SEM. n  =  6 /group at each time point. *: significant difference at p<0.05.

Changes in the body and muscle weights and muscle protein contents of HSF1-null and wild-type mice are shown in Figure 1. Although the body weight in each type of mice was identical between the 2nd and 4th week, that of wild-type mice was constantly higher than that in HSF1-null mice (p<0.05). There was no significant difference in the absolute muscle wet weight and proteins content of the contralateral control soleus muscle between wild-type and HSF-null mice. In wild-type mice, both muscle wet weight and protein content were significantly increased by 2 weeks of overloading, compared with the values of contralateral control (p<0.05). On the other hand, there was no significant effect of 2-week overloading on muscle weight and protein content in HSF1-null mice. Significant increase in wet weight and protein content in overloaded muscle was observed in wild-type and HSF1-null mice following 4 weeks of overloading (p<0.05). Increment of wet weight and protein content in overloaded soleus muscle relative to the contralateral control was significantly higher in wild-type mice than that in HSF1-null mice (Fig. 2, p<0.05).

### Fiber CSA

**Figure 3:**
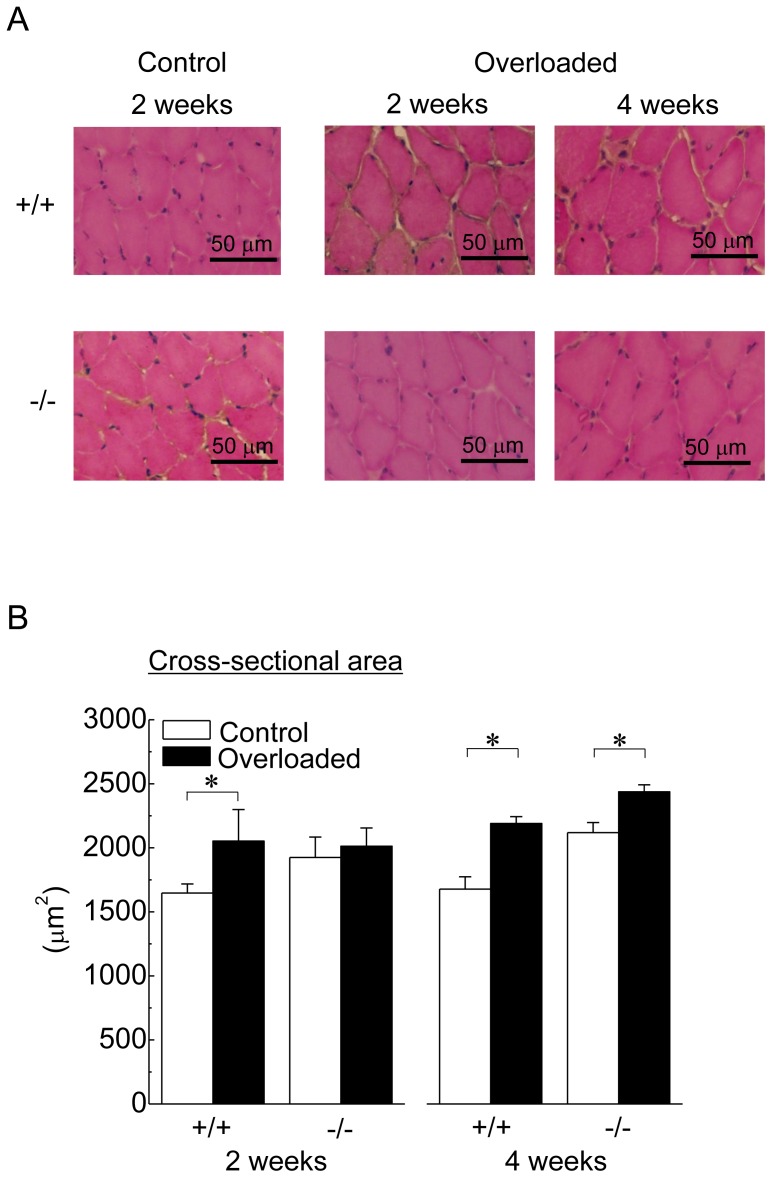
A: Transverse cryosections of the midbelly region of mouse soleus muscle stained with haematoxylin and eosin (H&E). B: Responses of the mean fiber cross-sectional area. See figure 1 for other abbreviations. Values are means ± SEM. n  =  6 /group at each time point. *: significant difference at p<0.05.

Figure 3A shows the representative cross-sectional images of soleus muscle in response to 2 and 4 weeks of overloading. Although there were no regenerating and damaged fibers in control and overloaded soleus muscle of both wild-type and HSF1-null mice, muscle fiber hypertrophy was noted in overloaded muscles.

Responses of mean fiber CSAs during the experimental period are shown in Figure 3B. There was no significant difference in fiber CSA of control soleus muscle between wild-type and HSF1-null mice. Mean fiber CSAs in wild-type mice were significantly increased by 2 and 4 weeks of overloading (p<0.05). Even though a significant increase in fiber CSA was observed following 4 weeks of overloading in HSF1-null mice (p<0.05), significant hypertrophy was not induced following 2 weeks of overloading.

### Pax7-positive nuclei

**Figure 4:**
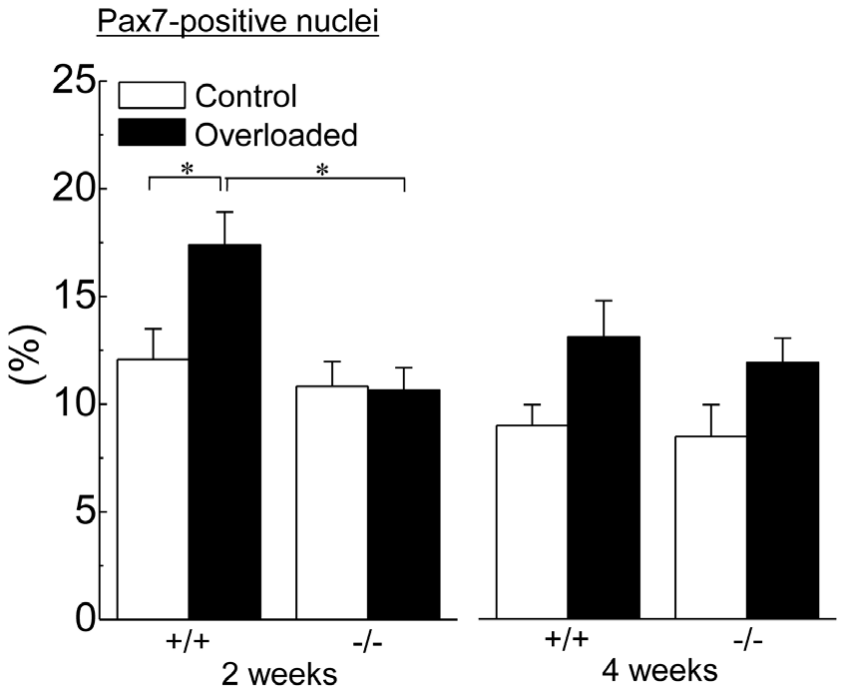
Changes in the population of Pax7-positive nuclei relative to the total myonuclei in response to functional overloading. Values are means ± SEM. n  =  6 /group at each time point. *: significant difference at p<0.05.

Figure 4 shows the changes in the distribution of Pax7-positive nuclei relative to total myonuclei in response to 2 and 4 weeks of overloading. There was no significant difference in the population of Pax7-positive nuclei of the contralateral control muscle between wild-type and HSF1-null mice. The population of Pax7-postive nuclei in wild-type mice was significantly increased after 2 weeks of overloading (p<0.05), but not in HSF1-null mice. The population of Pax7-positive nuclei in the overloaded muscle in wild-type mice was greater than that in HSF1-null mice (p<0.05). Following 4 weeks of overloading, insignificantly higher level of population of Pax7-positive nuclei was observed in both types of mice, compared with the level of control (p>0.05).

### HSF genes

**Figure 5:**
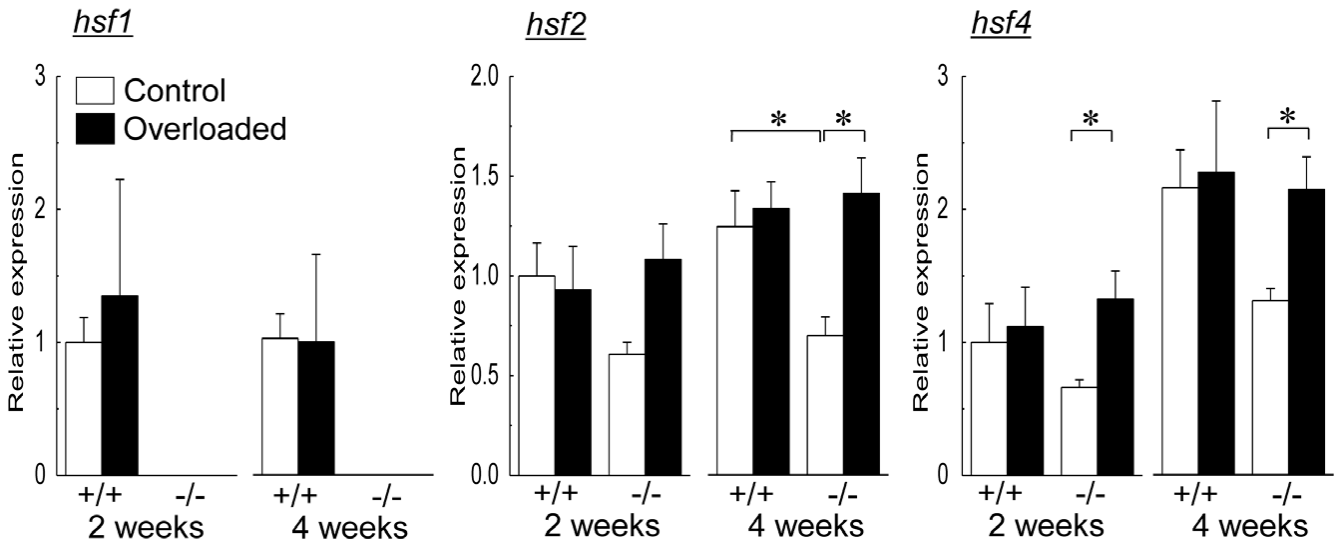
Changes in mean mRNA expression levels of heat shock transcription factor (HSF) 1, HSF2, and HSF4 in soleus muscle during the experimental period. *hsf1*, HSF1 mRNA; *hsf2*, HSF2 mRNA; *hsf4*, HSF4 mRNA. See figure 1 for other abbreviations. Values are means ± SEM. n  =  6 /group at each time point. *: significant difference at p<0.05.

Changes in mean mRNA expression levels of HSF1, HSF2, and HSF4 in soleus muscle during the experimental period are shown in Figure 5. HSF1 gene was not detected in HSF1-null mice. In wild-type mice, there were no significant changes associated with both growth and overloading in mRNA expression levels of HSF1, HSF2, and HSF4. On the other hand, significant increases were induced in mRNA expressions of HSF2 (4th week) and HSF4 mRNA (2nd and 4th week) by overloading compared with the contralateral control level in HSF1-null mice, respectively (p<0.05). Significantly different HSF2 mRNA expression in control muscles between wild-type and HSF1-null mice was noted at the 4th week (p<0.05).

### HSPs expressions

**Figure 6:**
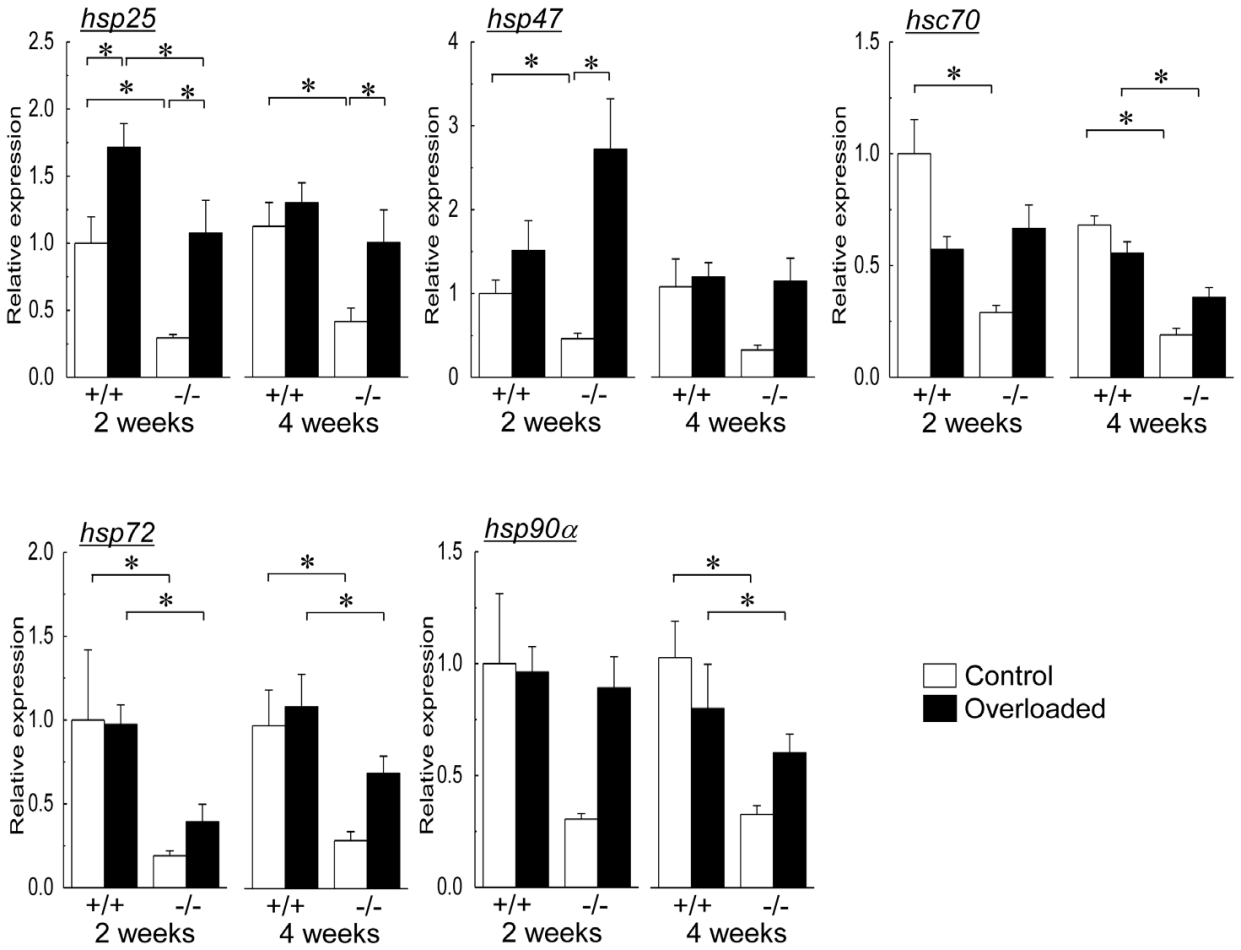
Changes in mean mRNA expressions of heat shock protein (HSP) 25, HSP47, HSC70, HSP72, and HSP90α. *hsp25*, HSP25 mRNA; *hsp47*, HSP47 mRNA; *hsc70*, HSC70 mRNA; *hsp72*, HSP72 mRNA; *hsp90α*, HSP90α mRNA. See figure 1 for other abbreviations. Values are means ± SEM. n  =  6 /group at each time point. *: significant difference at p<0.05.

The expression levels of HSPs were evaluated by real-time RT-PCR (at transcriptional level) and immunoblotting assay (at protein level). Changes in mRNA expressions of HSP25, HSP47, HSC70, HSP72, and HSP90α are shown in Figure 6. Absence of HSF1 gene (−/−) decreased the expression levels of HSP25 (2nd and 4th week), HSP47 (2nd week), HSC70 (2nd and 4th week), HSP72 (2nd and 4th week), and HSP90α (4th week) in the contralateral control soleus muscle versus those of wild-type mice (+/+, p<0.05).

In both wild-type and HSF1-null mice, mRNA expression level of HSP25 increased following 2 weeks of overloading (p<0.05, Fig. 6). After 4 weeks of overloading, significant up-regulation of HSP25 mRNA was still noted in HSF1-null (p<0.05), but not in wild-type, mice. The levels of HSP25 mRNA expression in the overloaded muscles in wild-type mice were significantly greater than in HSF1null mice after 2 weeks of overloading (p<0.05). HSP47 mRNA expression was significantly up-regulated in HSF1-null mice following 2 weeks of overloading (p<0.05), but not in wild-type mice. There were no significant changes in mRNA expression level of HSP47 in both types of mice after 4 weeks of overloading. Overloading-associated up-regulation was not inducted in mRNA expressions of HSC70, HSP72, and HSP90α in both wild-type and HSF1-null mice.

**Figure 7:**
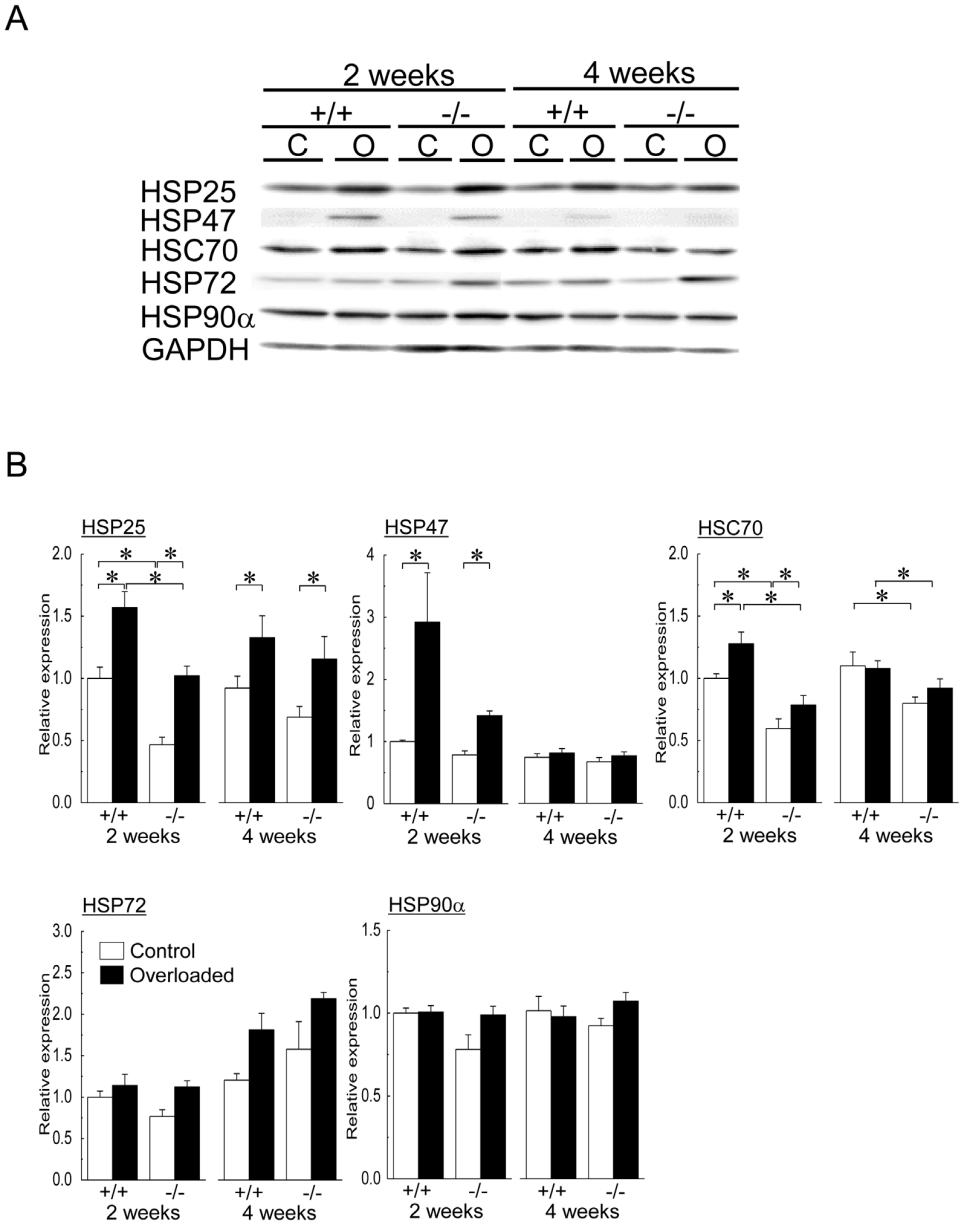
Expressions of heat shock proteins (HSPs) and heat shock cognate protein (HSC) in soleus muscle. A: Representative protein expression patterns of HSP25, HSP47, HSC70, HSP72, HSP90α and glyceraldehyde-3-phosphate dehydrogenase (GAPDH). C: control muscle, O: overloaded muscle. B: Changes in the mean expression levels of HSP25, HSP47, HSP70, HSP72, and HSP90α proteins. See figure 1 and 6 for other abbreviations. Values are means ± SEM. n  =  6 /group at each time point. *: significant difference at p<0.05.

Representative expression patterns of HSP25, HSP47, HSC70, HSP72, HSP90α and GAPDH proteins are shown in Figure 7A. Protein expression levels of HSP25 (2nd week) and HSC70 (2nd and 4th week) of the contralateral control soleus muscle in HSF1-null mice were significantly lower than those in wild-type mice (Fig. 7B, p<0.05). Significant increases in protein expression levels of HSP25, HSP47, and HSC70 were observed in both wild-type and HSF1-null mice following 2 weeks of overloading (p<0.05). The mean expression levels of HSP 25 and HSC70, but not of HSP47, in wild-type mice were significantly higher than those in HSF1-null mice following 2 weeks of overloading (p<0.05).

Increased expression levels of HSP25 protein were also observed in both wild-type and HSF1-null mice following 4 weeks of overloading (Fig. 7B, p<0.05). The mean expression level of HSC70 protein in wild-type mice was significantly higher than that in HSF1-null mice (p<0.05). There was no significant difference in the protein expression levels of HSP47 following 4 weeks of overloading. Overloading-related changes in protein expression levels of HSP72 and HSP90α were not observed in both HSF1-null and wild-type mice, either.

### Akt levels

**Figure 8:**
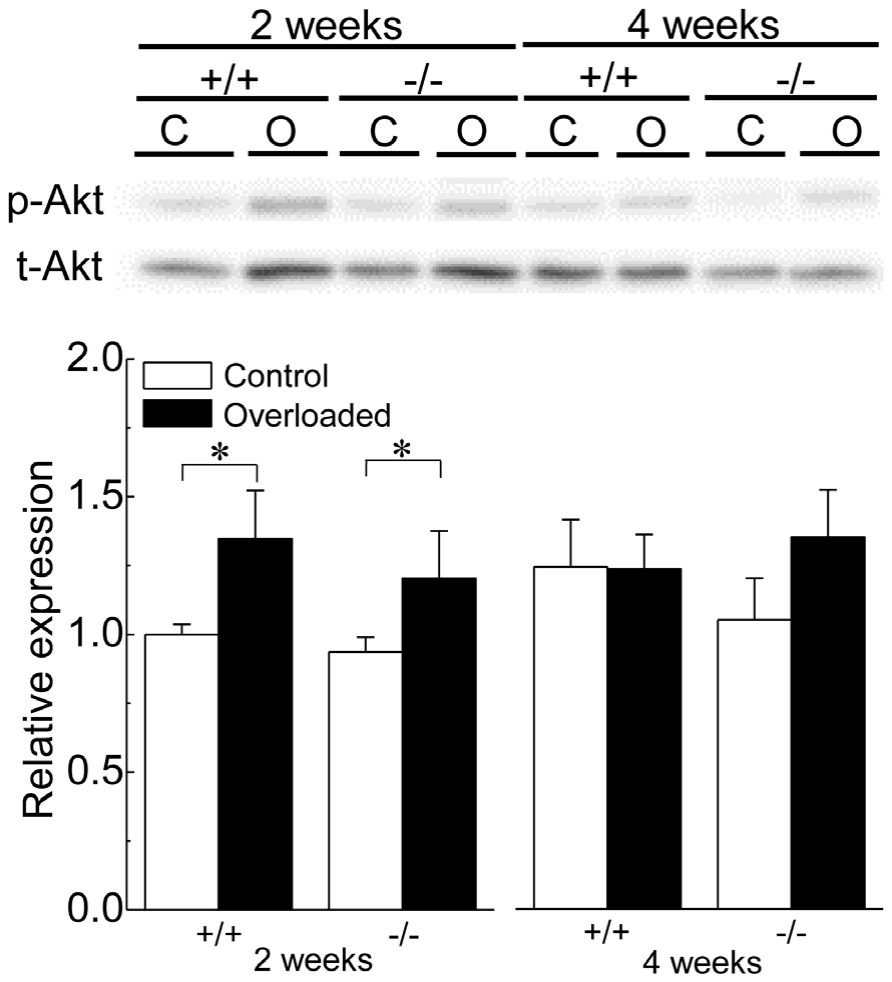
Changes in the expression level of Akt protein in soleus muscle. p-Akt: phosphorylated form of Akt (protein kinase B), t-Akt: total protein of Akt. See figure 1 and 7 for other abbreviations. Values are means ± SEM. n  =  6 /group at each time point. *: significant difference at p<0.05.

Figure 8 shows the representative responses of phosphorylated Akt (p-Akt), total Akt (t-Akt), and the mean level of p-Akt relative to t-Akt (p-Akt/t-Akt). Relative expression levels of p-Akt to t-Akt in control muscle were similar between wild-type and HSF1-null mice. Significant increases in the relative expression of p-Akt were observed in both wild-type and HSF1-null mice following 2, but not 4, weeks of overloading (p<0.05).

### Pro-inflammatory cytokines

**Figure 9:**
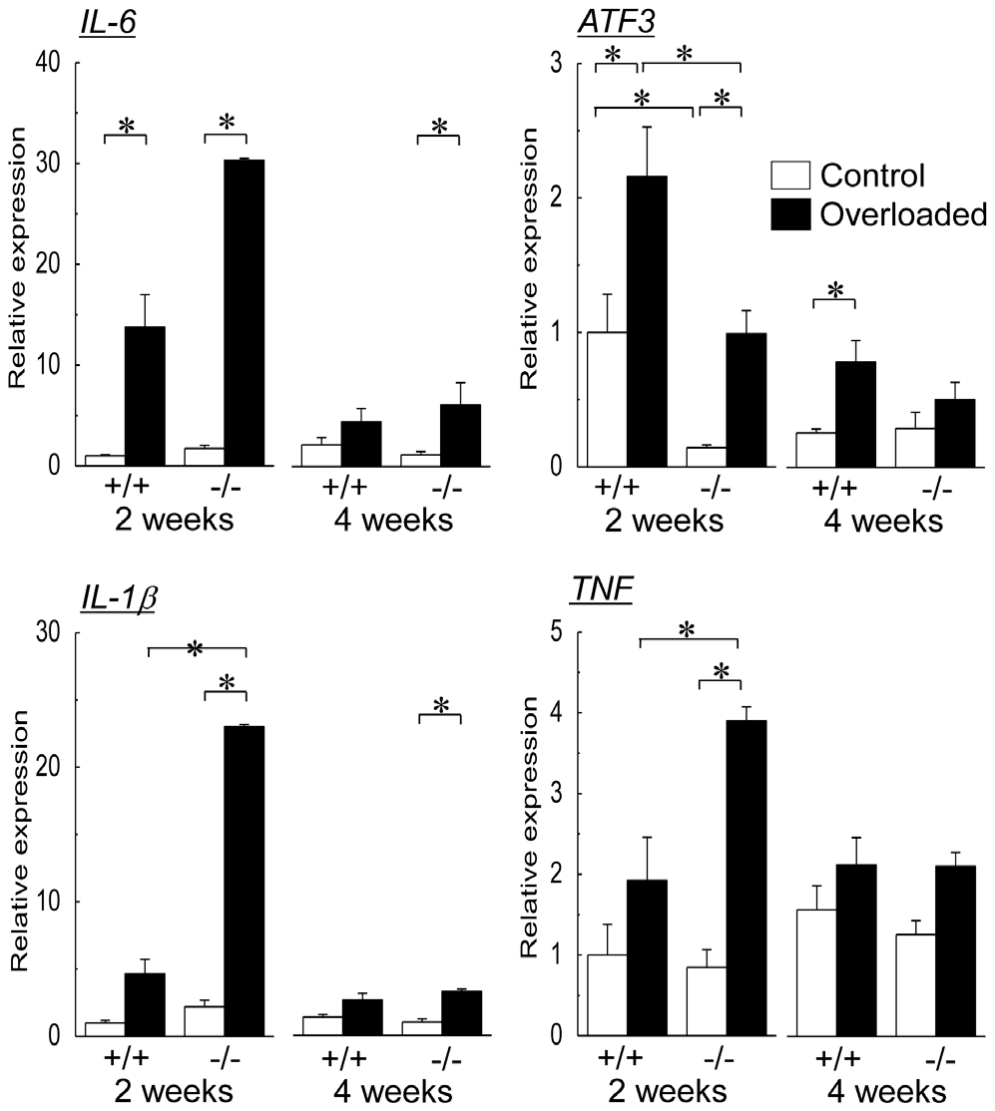
Changes in mean mRNA expression levels of pro-inflammatory cytokines in soleus muscle. *IL-6*, interleukin-6; *ATF3*, activating transcription factor 3; *IL-1β*, interleukin1β; *TNF*, tumor necrosis factor. See figure 1 for other abbreviations. Values are means ± SEM. n = 6/group at each time point. *: significant difference at p<0.05.

Changes in mean mRNA expression levels of pro-inflammatory cytokines, IL-6, ATF3, IL-1β, and TNF, in soleus muscle during the experimental period are shown in Figure 9. The levels of ATF3, but not of IL-6, IL-1β, and TNF, in control muscle of wild-type mice at the second week of experimental period was greater than those of HSF1-null mice (p<0.05). Significant up-regulations of IL-6 and ATF3 mRNAs were observed in both wild-type and HSF1-null mice following 2 weeks of overloading (p<0.05). The expression level of AFT3 mRNA in overloaded muscle of wild-type mice was greater than of HSF1-null mice (p<0.05). Significant up-regulation of IL-6 mRNA in HSF1-null mice, but not in wild-type, was still observed after 4 weeks of overloading (p<0.05). On the other hand, the overloading-related up-regulation of ATF3 mRNA expression was maintained in wild-type mice (p<0.05), but not in HSF1-null mice, after 4 weeks of overloading.

Expression levels of IL-1β and TNF mRNAs were also up-regulated in HSF1-null, not in wild-type, mice following 2 weeks of overloading (p<0.05, Fig. 9). The expression levels of IL-1β and TNF in the overloaded soleus muscle of HSF1-null mice were greater than in wild-type mice (p<0.05). Significant up-regulation of IL-1β mRNA expression was still observed in HSF1-null mice following 4 weeks of overloading, but not in wild-type mice (p<0.05).

## Discussion

Present study showed that absence of HSF1 partially inhibited the overloading-associated increments of muscle wet weight, protein content, and fiber CSA of mouse soleus muscle. This is the first report showing the inhibitory effects of HSG1-deficiency on skeletal muscle hypertrophy. Overloading-induced increase of the population of Pax7-positive muscle satellite cells, which may play a key role in skeletal muscle hypertrophy, was also attenuated by HSF1-defiency. On the other hand, HSF1 did not affect overloading-related phosphorylation of Akt. HSF1-deficiency decreased HSP expressions at mRNA and protein levels. Overloading-related up-regulations of not only HSF2 and HSF4 but also HSPs were observed in HSF1-deficient mice. Significant up-regulations of IL-1β and TNF mRNAs were observed in HSF1-null, not wild-type, mice following 2 weeks of overloading. Overloading-related increases of IL-6 and AFT3 mRNA expressions seen after 2 weeks of overloading tended to decrease after 4 weeks in both types of mice. In HSF1-null mice, however, the significant overloading-related increase in the expression of IL-6, not ATF3, mRNA was noted even at 4th week. Inhibition of muscle hypertrophy might be attributed to the greater and prolonged enhancement of IL-6 expression.

### Effects of HSF1-deficiency on overloading-associated skeletal muscle hypertrophy

In the present study, HSF1-deficiency did not affect the population level of Pax7-positive satellite cells in the control muscle. This result is consistent with our previous study [23]. But the overloading-associated increases of Pax7-positive satellite cells were inhibited in HSF1-null mice with restrained muscle hypertrophy. Although molecular mechanism(s) for HSF1-mediated regulation of satellite cells remains unclear, HSF1 may play a part in the regulation of proliferative potential of Pax-7-associated muscle satellite cells in overloading-induced skeletal muscle hypertrophy.

It is still unclear whether the level of Pax7-positive satellite cells affects overloading-associated skeletal muscle hypertrophy or not. A conditional knock-out technique revealed that satellite cell depletion did not inhibit the regrowth of skeletal muscle from unloading-induced atrophy [40]. This report also suggested the regrowth of atrophied soleus did not require the addition of new nuclei, which source is considered to be satellite cells. It was reported that muscle satellite cells were not essential for mechanical loading-associated skeletal muscle [41]. However, it was also suggested that the increased myonuclei in hypertrophied muscle fibers were attributed to muscle satellite cells, since the increase of myonuclei was observed in overloading-associated muscle hypertrophy under physiological condition [41]. On the contrary, another report indicated that the rate of regrowth in mouse soleus muscle was proportional to the level of Pax7-positive satellite cells [2].

In the present study, lower regrowing rate of overloaded skeletal muscle in HSF1-null mice was observed, compared with wild-type mice. On the other hand, a significant increase in the population of Pax7-positive satellite cells in wild-type mice was observed 2 weeks of overloading, but not in HSF-null mice. Therefore, greater regrowing rate in wild-type mice might be attributed to higher level of Pax7-positive satellite cells. However, the Pax7-positive satellite cells did not increase in HSF-null mice, even though muscle hypertrophy was observed following 4, but not 2, weeks of overloading. Physiological significance of the overloading-associated increase of Pax7-positive satellite cells is still unclear.

Mechanical overloading, as well as heat stress, has a stimulating effect on not only proliferative potential of skeletal muscle [2], [33], [35], [42] but also up-regulations of HSPs [13], [15], [43]. However, it is still unclear whether overloading-associated skeletal muscle hypertrophy is attributed to the chaperonic action of HSPs or stress-associated stimulation of protein synthesis. Although HSF1-deficiency modified the expressions of HSPs at mRNA and protein levels in the present study, the changes in HSP mRNAs were inconsistent with those in HSP proteins. These results were consistent with those in the previous study [16]. It has been also suggested that post-translational regulation plays a role in the regulation of HSP protein levels in skeletal muscle [44]−[46].

Lower expressions of HSP25 and HSC70 proteins in HSF1-deficient mice were noted in the current study. Overloading-associated up-regulations of HSP25 and HSC70 proteins were also attenuated by HSF1-deficiency. It has been reported that overexpression of HSP25 or HSP70 inhibits the immobilization-induced muscle atrophy via attenuation of increase in muscle-specific E3 ubiquitin ligases during disuse [10], [11]. In addition, drug-induced up-regulation of HSP72 stimulated differentiation and protein synthesis in C2C12 myoblasts [47]. Impaired regeneration of skeletal muscle related to deficiency of HSF1 [23] or inducible HSP70 [48] was also reported. Although there is no report regarding the effects of lower expression levels of HSP25 and HSC70 on skeletal muscle hypertrophy, lower expressions of these HSP proteins may be one of the causes for the inhibition of skeletal muscle hypertrophy.

HSF1-deficiency did not affect the expression level of HSP47 protein in normal soleus muscle. Up-regulation of HSP47 protein in association with muscle hypertrophy was noted regardless of the presence of HSF1 gene in the resent study. Physiological role of collagen-specific HSP47 in skeletal muscle hypertrophy is not unclear. Up-regulations of HSP47 proteins have been reported during regeneration of injured mouse soleus muscle [23] and in soleus muscle of rats exposed to hypergravitational environment [18]. HSP47, as well as HSP72 and HSP90α, may not have any role(s) in overloading-associated skeletal muscle hypertrophy, since there were no changes in the expression levels of HSP72 and HSP90α proteins.

### Possible effects of HSF1 on pro-inflammatory cytokines during skeletal muscle hypertrophy

In the present study, up-regulation of pro-inflammatory cytokines was observed following 2-week of overloading on soleus muscle. Previous studies showed that pro-inflammatory cytokines were up-regulated by increased workload, such as muscle contraction and exercise [25], . The present study demonstrated that expression levels of IL-6 and ATF3 mRNAs were up-regulated regardless of the presence of HSF1 gene. However, prolonged up-regulation of IL-6 mRNA by overloading was observed HSF1-deficient mice. On the contrary, overloading-associated up-regulation of ATF3 mRNA was enhanced by the presence of HSF1. Overloading-associated up-regulation of IL-6 may be depressed by HSF1-associated up-regulation of ATF3 [24] in skeletal muscle. Although it has been suggested that IL-6 plays a significant role in muscle satellite cell-mediated skeletal muscle hypertrophy [54], [55], the role(s) of the prolonged up-regulation of IL-6 and ATF3 in overloading-associated skeletal muscle hypertrophy remains unclear.

In the present study, HSF1-deficiency also enhanced the up-regulation of TNF and IL-1β mRNAs in response to overloading. As IL-1β and TNF function as the inhibitors of myogenic differentiation [27]−[29], enhanced up-regulations of these pro-inflammatory cytokines may be one of the causes for the inhibition of overloading-associated skeletal muscle hypertrophy in HSF1-null mice. Although TNFα signaling impairs insulin/insulin-like growth factor signaling by Akt [30], [31], a significant effect of presence or absence of HSF1 gene in p-Akt level was not observed. Further studies are essential to elucidate the regulatory roles of pro-inflammatory cytokines in overloading-associated skeletal muscle hypertrophy.

### Effects of HSF1-deficiency on the expression level of HSPs in response to overloading

Up-regulation of HSPs in mammalian skeletal muscle is mediated by HSF1 [21], [22]. However, up-regulations of HSPs were observed in both wild-type and HSF1-deficient mouse skeletal muscles in the present study. It was confirmed that HSF2 and HSF4 in HSF1-null mouse soleus muscle were up-regulated by 2 or 4 weeks of functional overloading, but not in wild-type mice. The up-regulation of these HSFs was also observed in response to reloading of atrophied soleus muscle in HSF1-null mice [16]. HSPs could be up-regulated by mechanical loading via HSF2 and/or HSF4, even if HSF1 gene is absent.

HSF2 content in cultured C2C12 mouse myoblasts (or myotubes) is transiently up-regulated by initiation of myogenic differentiation, suggesting that HSF2 may play a role in skeletal muscle development [56]. Both significant increase in muscle weight and HSF2 mRNA expression of overloaded soleus muscle in HSF1-null mice were observed following 4 weeks of overloading. Therefore, it is speculated that up-regulation of HSF2 might be a phenomenon caused as the compensation for HSF1-deficinecy, and cause the HSF2-dependent muscle hypertrophy in HSF1-null mice following 4 weeks of overloading.

Although expression of HSF4 mRNA is confirmed in skeletal muscle [57], HSF4 protein is detected in C2C12 mouse myoblasts, but not in B6D2F1 (BDF1) hybrid mouse skeletal muscle [58]. Even though human HSF4 functions to repress the expression of endogenous HSP27, HSP70, and HSP90 genes [57], there is no report concerning a physiological role of HSF4 in skeletal muscle. Up-regulations of HSF4 might be also a compensation for HSF1-deficinecy. Further studies are needed to elucidate a physiological role of HSF2 and HSF4 in skeletal muscle hypertrophy.

In conclusion, HSF1-deffiency inhibited overloading-associated skeletal muscle hypertrophy in mice. Loading-related-increase in Pax7-positive satellite cells was also attenuated by absence of HSF1. Inhibition of muscle hypertrophy in HSF1-null mice may be related to the enhanced up-regulation of pro-inflammatory cytokines, such as IL-6, IL-1β, and TNF, that act as inhibitors on myogenic differentiation. In addition, up-regulations of HSPs were inhibited in mice without HSF1 gene. HSF1 and/or HSF1-mediated stress response may play a key role in the overloading-associated skeletal muscle hypertrophy.
